# In this Issue: Volume 112, Issue 10, October 2021

**DOI:** 10.1111/cas.15135

**Published:** 2021-10-01

## Clonal hematopoiesis and associated diseases: a review of recent findings

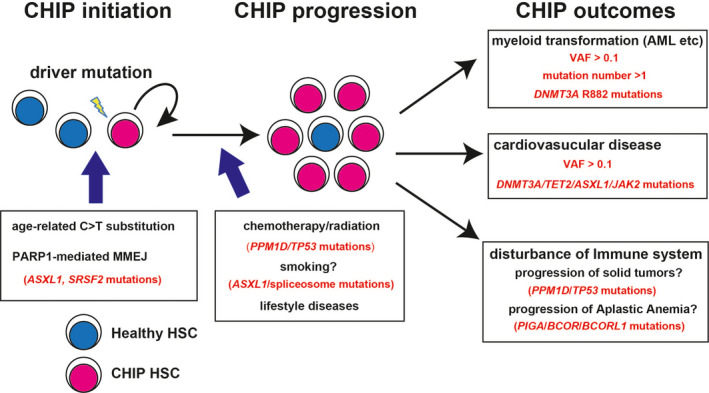

Cardiovascular diseases (CVDs) and cancers are among the leading causes of death in developed countries. These diseases have been linked to chronic inflammation, but the connection is not fully understood. Clonal hematopoiesis of indeterminate potential (CHIP), also frequently called as clonal hematopoiesis (CH), is clonal expansion of hematopoietic cells that are derived from hematopoietic stem cells (HSCs) with at least one driver mutation. CHIP has been linked to CVDs, hematologic diseases, solid tumors, and other non‐malignant diseases like aplastic anemia. CHIP is now extensively studied, and close association between CHIP and inflammation has been indicated. In this review, Asada and Kitamura cover the recent literature on CHIP. They discuss the how CHIP further propagates inflammation and creates a positive feedback loop. They also cover how CHIP‐associated mutations alter the functions of HSCs and immune cells. Our understanding of CHIP is still limited, but with further research we may be able to target these mutations and improve outcomes for a wide variety of diseases.

https://onlinelibrary.wiley.com/doi/full/10.1111/cas.15094

## Immunosuppressive activity is attenuated by astragalus polysaccharides through remodeling the gut microenvironment in melanoma mice

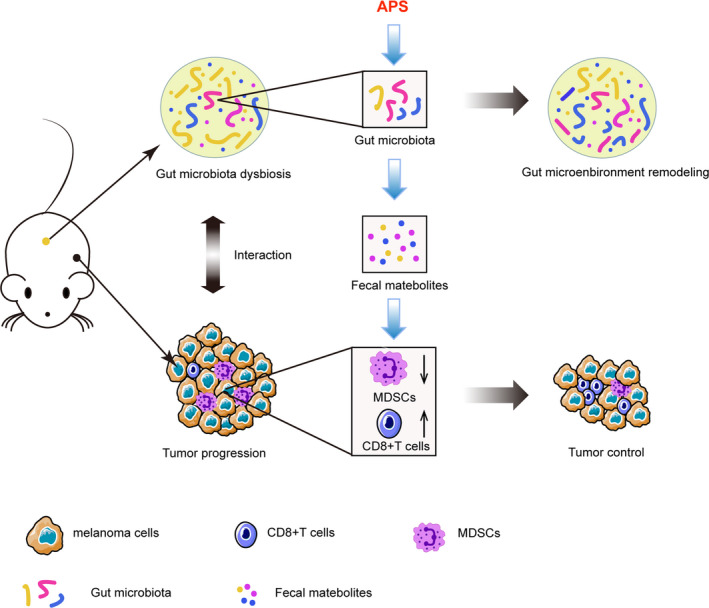

More and more research is showing the importance of the tumor microenvironment (TME) on tumor progression as well as treatment response. From intestinal malignancies, it is clear that the gut microbiome also plays a role in cancer growth and proliferation. There has been a paucity of data investigating the link between the gut microbiome and the TME. Ding et al have previously shown that *Astragalus polysaccharides* (APS) has anti‐tumor properties in melanoma‐bearing mice. In this study, Ding et al explored the possible underlying mechanisms by which APS inhibits tumor growth. They found that APS administration downregulates the population of immunosuppressive myeloid‐derived suppressor cells (MDSCs). They also showed that APS altered the composition of bacteria in the gut microbiota in the mouse model, which subsequently attenuates the immunosuppressive activity of MDSCs. This altered metabolite profile may be the link between the gut microbiome and the TME.

https://onlinelibrary.wiley.com/doi/full/10.1111/cas.15078

## Alantolactone is a natural product that potently inhibits YAP1/TAZ through promotion of reactive oxygen species accumulation

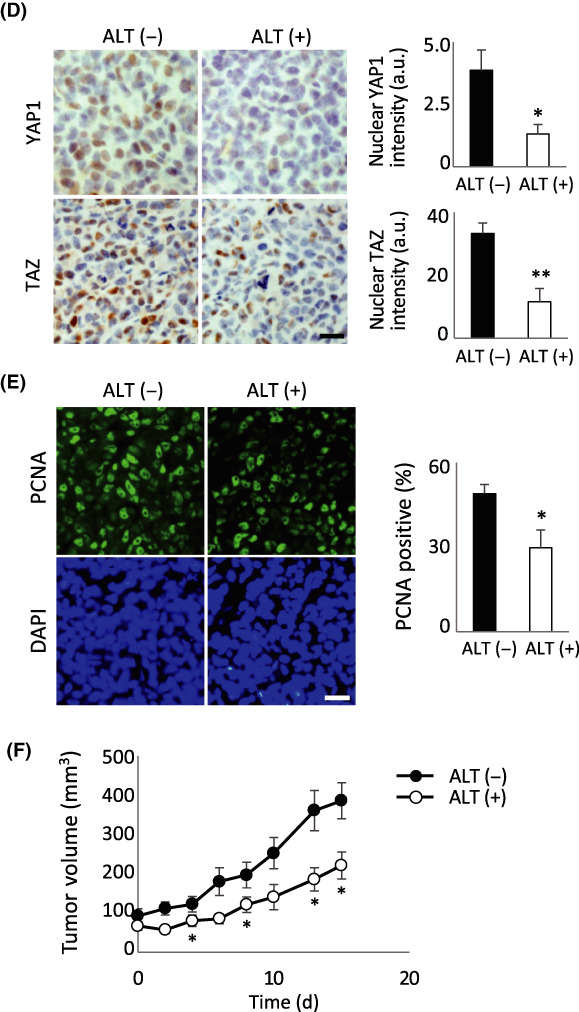

Yes‐associated protein 1 (YAP1) pathway is elevated in a majority of malignancies. This makes YAP1 and its paralogue Transcriptional coactivator with PDZ‐binding motif (TAZ) critical targets for novel therapeutics. In this study, Nakatani et al screened a large chemical compound library to find potential YAP1/TAZ inhibitors and found alantolactone (ALT), a sesquiterpene lactone, to be a promising candidate. They were able to show that ALT blocked cancer progression in vivo. They found that mice treated with ALT had reduced YAP1 levels and that this effect could be reversed with NAC, a reactive oxygen species (ROS) scavenger. ALT acts on many different pathways, but these data suggest that its main anti‐tumor activity depends on the ROS generation via thioredoxin reductase (TrxR) inhibition. Further studies into antioxidant inhibitors that target the YAP1 pathway may lead to significant advancements in cancer care.

https://onlinelibrary.wiley.com/doi/full/10.1111/cas.15079

